# Characterization and Evolutionary Analysis of Non-Canonical Heat Shock Protein 70 Family Members in Metazoan

**DOI:** 10.3390/ijms262311363

**Published:** 2025-11-24

**Authors:** Jiabo Tan, Xiaohan Li, Qi Wang, Weiqi Xu, Jixiang Liu, Yunlong He, Wenhui Yin, Jiahao Li, Xinyu Li, Xiaojun Song, Kefeng Xu, Guodong Wang

**Affiliations:** 1 School of Marine Science and Engineering, Qingdao Agricultural University, Qingdao 266109, China; 2 Marine Science Research Institute of Shandong Province, National Oceanographic Center, Qingdao 266104, China; 3 Fisheries College, Ocean University of China, Qingdao 266100, China

**Keywords:** heat shock protein 70, non-canonical, cellular and molecular function, conserved motif, evolutionary

## Abstract

To broaden the scope of research on the characteristics and evolutionary relationships within the heat shock protein 70 (Hsp70) family, encompassing its non-canonical members, amino acid sequences of Hsp70-12, Hsp70-13, and Hsp70-14, alongside those of traditional Hsp70, were collected and analyzed. The findings indicate that, during the evolution of metazoans, the various Hsp70 groups diverged from one another. Specifically, Hsp70-12 emerges as the least conserved member, as evidenced by structural alignment data and the Ka/Ks ratio. It not only represents the most distantly related group to traditional Hsp70 but also stands out as the sole alkaline group within the family. In contrast, Hsp70-13 exhibits a close evolutionary relationship with traditional Hsp70, albeit with the notable loss of its C-terminal domain. Hsp70-14 occupies an intermediate position between Hsp70-12 and Hsp70-13. Phylogenetic analysis suggests that these groups diverged prior to the advent of invertebrates. Furthermore, five conserved motifs within the ATP-binding domain of Hsp70, which serve as distinguishing features for Hsp70 groups, were identified. The diverse characters of the non-canonical Hsp70s are probably related to their special cellular location and tissue specificity. Together, the results of this research will help identify and categorize Hsp70s. Further research that aims at identifying additional non-canonical Hsp70 members and elucidating the distinct characteristics and functions of these molecular chaperones will enhance our comprehension of the origin and evolutionary trajectory of the Hsp70 family.

## 1. Introduction

The 70 kDa heat shock proteins (Hsp70s) constitute a highly conserved protein family that has been identified across a wide range of prokaryotic and eukaryotic species [1,2]. As pivotal molecular chaperones within cells, Hsp70s play indispensable roles in numerous cellular processes. These include, but are not limited to, the refolding of naïve or misfolded proteins, facilitating the transport of newly synthesized protein through membrane structures, recruiting proteins to the proteasome, and assisting in the chaperone-mediated autophagy [3]. The cellular functions of Hsp70s enable them to actively participate in responses to various environmental stressors, such as extreme temperatures, osmotic pressure, ultraviolet light, and pathogenic infections [4,5,6,7]. Consequently, Hsp70s have been employed as biomarkers of environmental stress in both laboratory and field experiments [8].

Ever since the first discovery in *Drosophila*, Hsp70 has been widely found throughout the animal kingdom. Typically, each organism possesses multiple Hsp70s; for example, to date, thirteen Hsp70s have been identified in human beings and eleven in *Drosophila* [9]. The gene duplication events during evolution led to the emergence of multiple organelle-specific isoforms and diverse lineages of Hsp70s in eukaryotes [10]. Several attempts were made to classify and characterize different Hsp70 groups. For example, previous research has categorized metazoan Hsp70s into four lineages or clusters, cytosolic A and B, endoplasmic reticulum (ER), and mitochondria, based on their intracellular location [11,12]. Another study further divided cytosolic Hsp70s into eight clades [13]. Both classifications were supported by phylogenetic analyses and several genome-wide Hsp70 screening studies [10,13]. However, it is important to note that these classifications primarily focused on canonical members of Hsp70s. However, some so-called non-canonical members, such as Hsp70-12, Hsp70-13, and Hsp70-14, were not included.

Nearly all Hsp70 family members share a similar structural architecture, consisting of an N-terminal nucleotide-binding domain (NBD) followed by a substrate-binding domain (SBD) and a variable region [14]. In eukaryotic species, cytosolic Hsp70 typically terminates with a conserved motif (Glu-Glu-Val-Asp; EEVD), which is functional in interacting with Hsp70 cofactors [3,15]. In contrast, non-canonical Hsp70s do not exhibit these characteristics. To date, none of the identified non-canonical Hsp70s ends with EEVD. Members of individual eukaryotic subgroups exhibit higher similarity to each other, with cytoplasmic Hsp70 cluster members sharing at least 71% identity [11]. However, the structure of the non-canonical Hsp70s differs from that of traditional Hsp70s, with some non-canonical members lacking parts of conserved domains. For instance, Hsp70-13 has been found to lose the C-terminal substrate-binding domain during evolution [16].

The Hsp70 family comprises organelle-specific members localized in the cytosol, endoplasmic reticulum (ER), mitochondria, and chloroplasts, and it performs chaperone functions as well as being involved in the transfer of proteins along or through organelle membranes [7,17]. Recent studies on the non-canonical members revealed that they are localized to different cellular compartments and play various roles in cellular events such as apoptosis, proteostasis, and maintaining cell organ structure, similar to traditional Hsp70s [18,19,20]. For example, Hsp70-12 protects neurons by assisting in apoptosis [21]. Hsp70-13 is functional in the structural maintenance of the ER in epithelial cells and is implicated in cancer generation in various organs [17,18], and Hsp70-14 is essential in the formation of a lens during embryogenesis [20]. The search and characterization of Hsp70s revealed that non-canonical Hsp70s possess distinct structures and characteristics, which probably determine their specific locations and functions within tissues and cells. Our recent research identified Hsp70-12 and Hsp70-14 in the water flea *Daphnia magna*, revealing unique characteristics and functions distinct from the traditionally defined Hsp70 members [22].

In this study, we aim to characterize the non-canonical Hsp70 groups and elucidate their evolutionary relationship by simulating the domain structures and conducting further phylogenetic analyses of Hsp70s from representative species across metazoans. We provide essential information for identifying non-canonical Hsp70s and understanding their relationships with traditional members. The findings of this research offer new insights into the evolution of the Hsp70 family and will aid in the categorization of Hsp70s identified in the future.

## 2. Results

### 2.1. The Variation in Isoelectric Points and Molecular Weights

**Figure 1:**
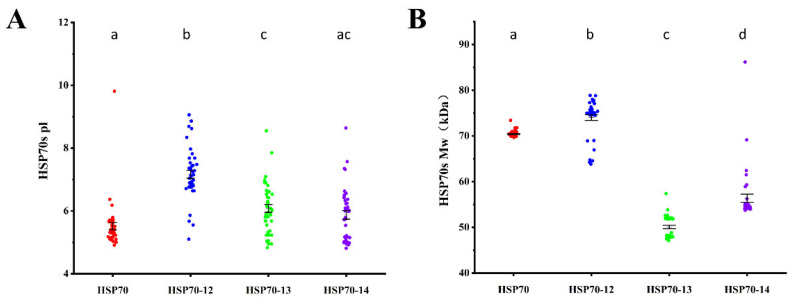
Molecular weight (MW) and isoelectric point (pI) distribution of Hsp70s in different groups. (**A**) Scatter plot of pI distribution. The average pI is 5.52, 7.17, 6.08, and 5.87 for Hsp70, Hsp70-12, Hsp70-13, and Hsp70-14, respectively. (**B**) Scatter plot of MW distribution. The average MW is 70.42 kDa, 74.01 kDa, 50.08 kDa, and 55.92 kDa for Hsp70, Hsp70-12, Hsp70-13, and Hsp70-14, respectively. Error bars represent standard deviation (SD). Groups sharing the same letters are not significantly different at the 5% level of significance.

The average putative isoelectric points (pIs) of Hsp70s, Hsp70-12s, Hsp70-13s, and Hsp70-14s are 5.52, 7.17, 6.08, and 5.87, respectively. Of the four groups, the most alkaline one is Hsp70-12 and the most acidic one is Hsp70 (Figure 1A). Hsp70-13 has the smallest predicted molecular weight (Mw), only 50.08 kDa, followed by Hsp70-14 (55.92 kDa), Hsp70 (70.42 kDa), and Hsp70-12 (74.01 kDa) (Figure 1B).

### 2.2. The Differences in 3D Structure

**Figure 2:**
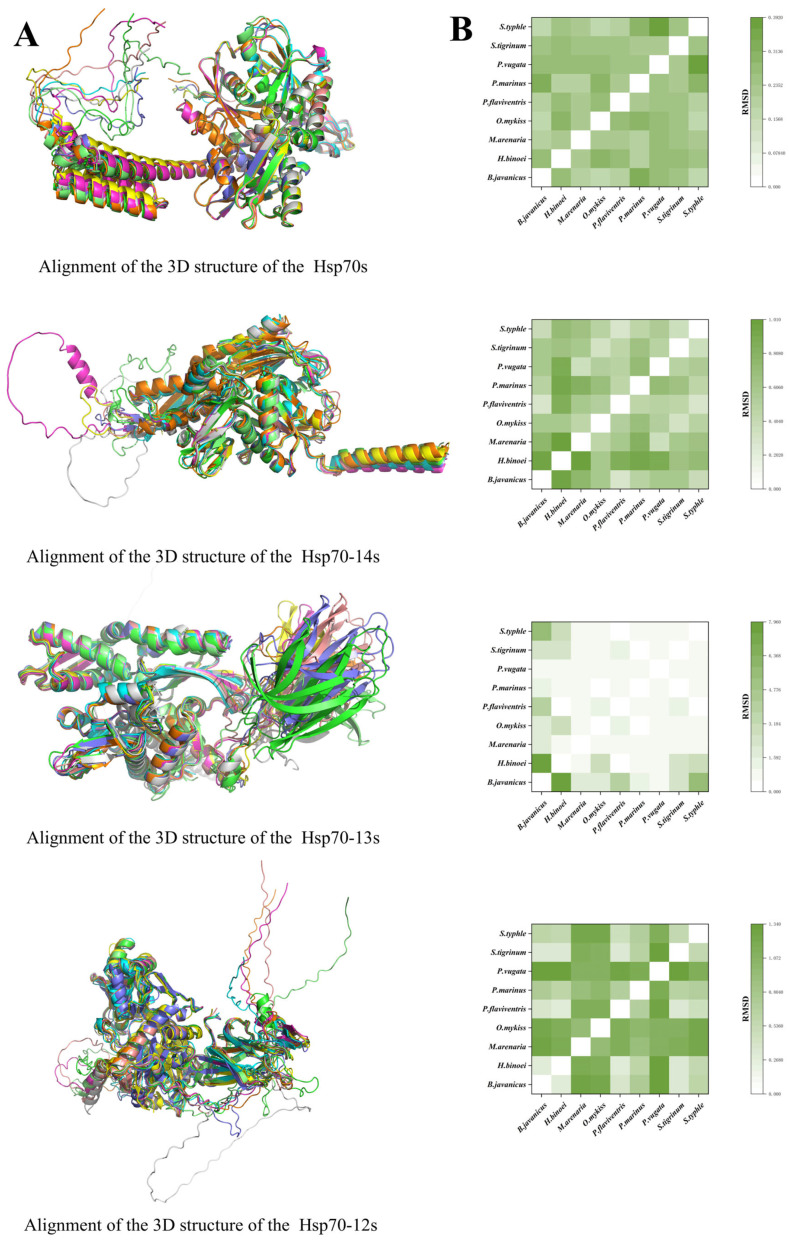
Alignment of the 3D structure of the Hsp70s and their RMSD values. (**A**) The alignment of the 3D structure of different Hsp70 groups simulated using AlphaFold3 (AlphaFold Server—Google DeepMind). Representative species with all four types of Hsp70s were selected, and the sequences were used for simulation and alignment. (**B**) The RMSD values of structural alignments of different Hsp70 groups. The green gradient represents different RMSD values. Darker color equals greater value. The values were calculated using PyMOL.

A total of 36 representative sequences from nine species for the Hsp70 groups were selected to build 3D models and perform further alignment (Figure 2). The mean intra-group RMSDs of the four groups were 1.27 Å, 0.89 Å, 0.59 Å, and 0.25 Å, with Hsp70-14 being the highest, followed by Hsp70-12, Hsp70-13, and Hsp70 (Figure 2).

### 2.3. The Conserved Motifs of Hsp70 Family Members

**Figure 3:**
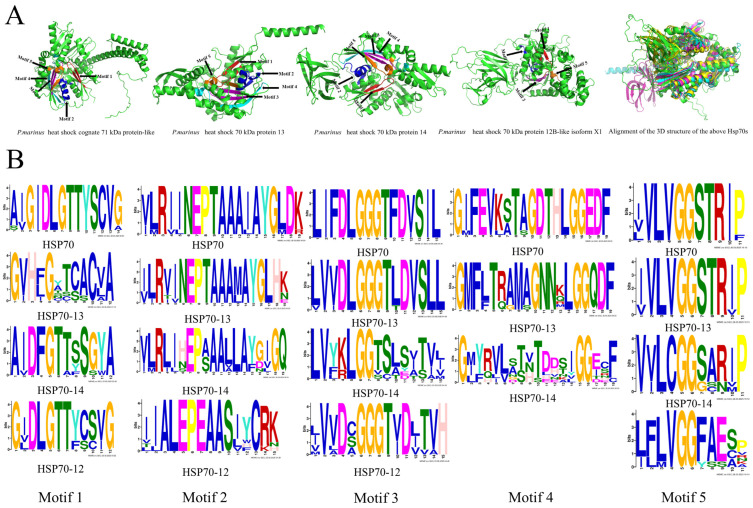
The sequences of conserved motifs in different Hsp70 groups and the predicted positional annotations of the motifs mapping into the 3D structures of *P. marinus* Hsp70s. (**A**) Conserved motifs in the 3D structure of *P. marinus* Hsp70s. Three-dimensional models were predicted using the alphafold3 software (AlphaFold Server—Google DeepMind). Colored regions represent sequence motifs. The red section represents Motif 1, the blue section represents Motif 2, the purple section represents Motif 3, the cyan section represents Motif 4, and the orange section represents Motif 5. (**B**) Conserved motifs in different Hsp70 groups. Online software MEME was used to search for motifs, and the motif sequences were presented using Prosite.

For the four different Hsp70 groups, five motifs were identified in the amino acid sequences, except Hsp70-12, which lacks motif 4 (Figure 3B). The conserved motifs have different characteristic amino acids: motif 1 is characterized by conserved “DxGTT”, except in Hsp70-13; motif 2 is characterized by “EPxAA”; motif 3 is characterized by “GGxT”; motif 4 is characterized by “16G17G”; and motif 5 is characterized by “LxGG” (Figure 3B). The motifs in Hsp70s are usually more conserved than in the other three groups of Hsp70s. Using the four Hsp70s from *P*. *marinus* (XP_032835511.1, XP_032806443.1, XP_032816507.1, and XP_032810070.1), the slight differences in the locations of the motifs are shown (Figure 3A).

### 2.4. Phylogenetic Analysis of Hsp70 Family Members

**Figure 4:**
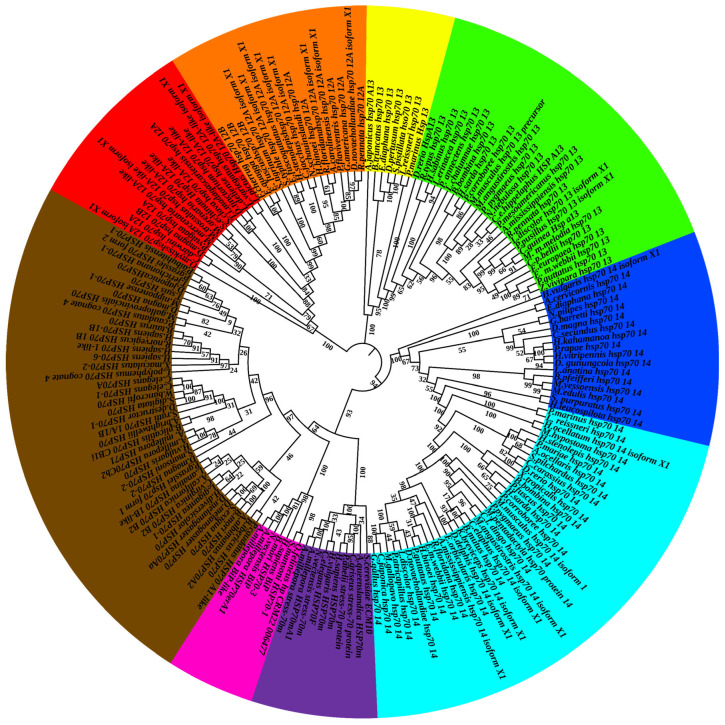
Phylogenetic tree classification of Hsp70s. The phylogenetic tree was constructed using the neighbor-joining (NJ) method with Hsp70 amino acid sequences collected from NCBI. The number represents the bootstrap value. Invertebrate Hsp70-12 (red), vertebrate Hsp70-12 (orange), invertebrate Hsp70-14 (yellow), vertebrate Hsp70-14 (green), invertebrate Hsp70-13 (light blue), vertebrate Hsp70-13 (blue), ER Hsp70 (magenta), mitochondrial Hsp70 (brown), and cytosolic Hsp70 (pink) are marked with different colors.

A total of 172 sequences of the Hsp70 family were collected from GenBank. The acquired sequences included 42 cytosolic Hsp70, 5 ER Hsp70, 9 mitochondrial Hsp70, 30 Hsp70-12A, 33 Hsp70-13, and 53 Hsp70-14. Effects were made to choose taxa covering diverse phyla (Supplementary Table S2). Based on the sequences collected and analyzed, Hsp70-13 is more closely related to Hsp70, followed by Hsp70-14, while Hsp70-12 is distant from the other three groups (Figure 4). In all three non-canonical Hsp70 groups, the ones from vertebrates and invertebrates form separate clades. For the Hsp70 group, the mitochondrial Hsp70, ER Hsp70, and cytosolic Hsp70 form clades on their own (Figure 4).

### 2.5. Analysis of Synonymous and Nonsynonymous Substitution Rates

**Figure 5:**
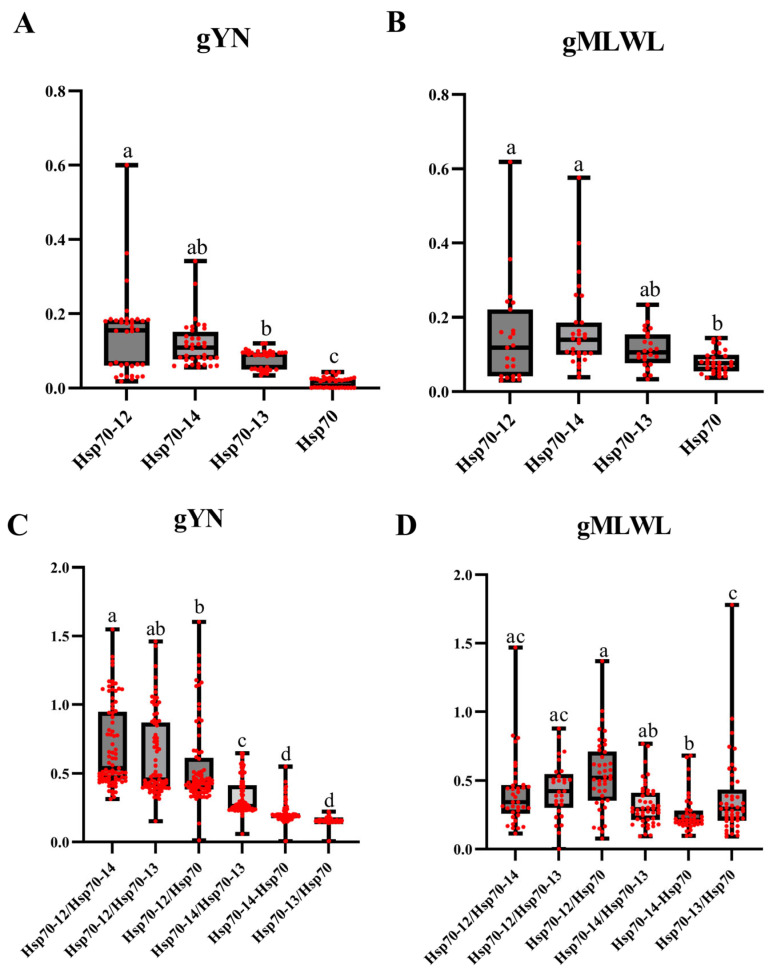
Inner- and inter-group Ka/Ks (ω) values of Hsp70s. Utilizing the KaKs_Calculator 2.0 software, the inner- and inter-group Ka/Ks values were calculated using the gYN and gMLWL methods. (**A**) Ka/Ks was calculated using the gYN method for each Hsp70 group. The average Ka/Ks were 0.142 (Hsp70-12), 0.118 (Hsp70-14), 0.079 (Hsp70-13), and 0.016 (Hsp70). (**B**) Ka/Ks was calculated using the gMLWL method for Hsp70 groups. The average Ka/Ks were 0.147 (Hsp70-12), 0.165 (Hsp70-14), 0.114 (Hsp70-13), and 0.079 (Hsp70). (**C**) Ka/Ks was calculated using the gYN method between each Hsp70 group pair. The average Ka/Ks were 0.694 (Hsp70-12/Hsp70-14), 0.621 (Hsp70-12/Hsp70-13), 0.546 (Hsp70-12/Hsp70), 0.33 (Hsp70-14/Hsp70-13), 0.207 (Hsp70-14/Hsp70), and 0.155 (Hsp70-13/Hsp70). (**D**) Ka/Ks was calculated using the gMLWL method between each Hsp70 group pair, and they were 0.411 (Hsp70-12/Hsp70-14), 0.435 (Hsp70-12/Hsp70-13), 0.54 (Hsp70-12/Hsp70), 0.321 (Hsp70-14/Hsp70-13), 0.259 (Hsp70-14/Hsp70), and 0.371 (Hsp70-13/Hsp70). The two end whiskers represent the maximum and minimum values, while the two ends of the box represent the upper and lower quartiles, respectively. The line in the middle of the box is the mean value. The Brown–Forsythe test and Bartlett’s test were employed to examine the significance. Groups sharing the same letters are not significantly different at the 5% level of significance.

The synonymous and nonsynonymous substitution rates (Ks and Ka) of 41 representative Hsp70 family members were calculated using gYN and gMLWL methods. The average Ka/Ks inside each group and between every two group pairs were calculated and presented (Figure 5). Of the four groups, the Hsp70 group has the lowest Ka/Ks ratio within-group, accompanied by the smallest variation. At the same time, both Hsp70-12 and Hsp70-14 groups have a great within-group Ka/Ks ratio using either calculation method. Despite the differences in the average number and level of variation, all four types of Hsp70 have a within-group Ka/Ks of less than one. As for the between-group comparison, the average pair-to-pair Ka/Ks ratios are less than one. Yet the numbers of Hsp70-12 with the other three groups are higher than the rest of the pairwise comparison numbers, but they are not significant using both methods (Figure 5).

## 3. Discussion

For now, the majority of studies on Hsp70s have predominantly centered on the traditionally recognized members of the family, with the origin and evolutionary trajectory being relatively well-established [13,23,24]. However, to our best knowledge, only a limited number of studies delved into the functions and characters of non-canonical Hsp70s, namely Hsp70-12, Hsp70-13, and Hsp70-14. In recent years, the continuing expansion of available genomic data has opened new avenues for us to extend our interest to the non-canonical Hsp70 members. Consequently, in the current research, we meticulously selected the amino acid sequences from species with all four types of full-length Hsp70s, and we identified and analyzed their characteristics to minimize interspecies differences.

Among the examined Hsp70s, as the member with the largest molecular weight, Hsp70-12 tends to exhibit a more alkaline nature, in contrast to the other three Hsp70 groups, which are acidic proteins. The phenomenon was described before in *D. magna* [22]. Hsp70-12 has a role in protecting animals against extreme stress, just like cytosolic Hsp70 [2,22]. But why Hsp70-12 is alkaline while the other Hsp70s are acidic is still not clear.

Regardless of their types, all signature motifs identified are located in the ATP-binding domain of Hsp70. The presence of these conserved motifs suggests that the ATPase domain is well-conserved across different Hsp70 groups. Considering the indispensable role of this domain in the functional process of Hsp70 chaperone activity, the finding is reasonable. The normal functioning of Hsp70 relies on the catalyzing of ATP, irrespective of the group [25,26]. Among the characterized Hsp70s in the current research, Hsp70-13 lacks a substrate-binding domain but still retains a functional ATP-binding domain containing all the Hsp70 signature motifs, thereby justifying its classification within the Hsp70 family. Therefore, gaining a deeper understanding of the characteristics and locations of the conserved motifs will further aid in the identification and classification of Hsp70s.

The observed variations in protein structure and characteristic motifs among the four Hsp70 groups may stem from their distinct cellular locations and functions. It is proposed that Hsp70 functions in both the cytoplasm and nucleus [27]. Specifically, Hsp70-12 is localized to the cytoplasm, nucleus, mitochondria, and endoplasmic reticulum, and it is consistently detected in endothelial cells during development or against environmental stress [19,22]. Moreover, Hsp70-12 is highly tissue-specific [19,28], being identified in the gonad, mantle, hepatopancreas, and gill, but not in other tissues and organs of the mussel *Mytilus galloprovincialis*, with the gonad displaying the highest transcript levels [29]. Meanwhile, Hsp70-13 is localized to the luminal side of microsome fractions and is consistent with an endoplasmic reticulum (ER) and other intraorganellar localization [16]. Previous studies demonstrated that Hsp70 subfamilies are distinguished by the C-terminal motifs, such as GP(T/K)(V/I)EE(V/M)D, KDEL, NUCDISC, and MITDISC, respectively. The listed motifs typically determine the subcellular localization of the Hsp70s [13,30,31]. However, this does not apply to non-canonical Hsp70s, as the three groups of non-canonical Hsp70s described in the current study lack conserved characteristic C-terminal motifs across different species. Consequently, the mechanisms by which these non-canonical Hsp70s apply to achieve proper cellular localization remain to be elucidated.

Phylogenetic analysis reveals that the members of the Hsp70 group are well-separated, with those from invertebrates and vertebrates clearly distinguished in the Hsp70-12, Hsp70-13, and Hsp70-14 groups. Structural and phylogenetic analyses suggest that Hsp70s likely originated from the same ancestor and underwent various mutations during evolution, leading to the formation of distinct groups. Non-canonical Hsp70s diverged after the emergence of metazoans and before the appearance of vertebrates, as evidenced by the division of all three non-canonical groups into invertebrate and vertebrate sub-groups (Figure 4). Following their divergence, non-canonical Hsp70s have been employed to fulfill similar yet distinct roles. The Hsp70-12 subfamily is the least conserved among Hsp70s, judged by primary sequence homology. This chaperone is expressed in endothelial cells and is required for angiogenesis, with its unblocked N-terminus being essential for these angiogenic activities [19]. Hsp70-12 also protects against endotoxin-induced cardiac dysfunction by activating the PI3K/Akt signaling pathway and plays specific neuronal protection roles along with assisting in neuronal apoptosis [21,32,33]. Collectively, these findings suggest that Hsp70-12 is expressed in ectoderm-related tissues and performs Hsp70-like functions. Hsp70-13 is the smallest Hsp70, due to the lack of the C-terminus peptide-binding domain [16,34]. Therefore, Hsp70-13 can be considered as a truncated version of canonical Hsp70. However, it still has an ATPase activity independent of peptide stimulation [16]. Despite the loss of the C-terminal part during evolution, Hsp70-13 remains closely related to canonical Hsp70 compared to other non-canonical Hsp70 groups studied in the current research. Hsp70-13 is functional in maintaining the homeostasis of cytosolic and secretory proteostasis [18]. In addition to Hsp70-13, Hsp70-14 also appears to have lost a certain sequence compared to canonical Hsp70 [16]. Notably, both Hsp70-13 and Hsp70-14 lack certain domains essential for canonical Hsp70 functions, but participate in the unfolded protein response, similar to canonical Hsp70 [16,18]. However, their roles are limited to specific cell structures within certain tissues during a few cellular and molecular events. For example, Hsp70-14 was proven to be essential in the formation of the zebrafish lens during embryogenesis [20]. Continuing studies on the functions and cellular locations of non-canonical Hsp70s will enhance our understanding of their origins and evolution.

In addition to phylogenetic analysis, the current study also calculated the Ka/Ks ratio both within and between Hsp70 groups. The results reveal that all Hsp70 groups exhibit a Ka/Ks ratio of significantly less than one, indicating that strong negative selections have acted upon the investigated Hsp70 groups. Furthermore, the average pair-to-pair Ka/Ks ratios between the Hsp70 groups are also less than one, suggesting that negative selections have occurred among these groups as well. Purifying selection was reported in mammalian, nematode, molluscan, and protist Hsp70 genes [24,35,36,37]. Our extended research into non-canonical Hsp70s aligns with these earlier findings. The phenomenon indicates a conservative role of the Hsp70 family within metazoans. More dramatically, traditional Hsp70 is under stronger purifying selection pressure compared to its non-canonical counterparts, implying that traditional Hsp70s are probably more critical in animals. Hsp70 is indispensable for animal survival. Knockout studies have demonstrated that mice lacking Hsp70 are more sensitive to stress, while the knockout of Hsc70, the constitutive homolog of Hsp70 in cells, has proven to be lethal [1,38]. Along with their co-chaperones, Hsp70s are involved in a multitude of cellular processes, including protein folding and quality control, assisting in the degradation of misfolded protein, anti-apoptosis, mediating autophagy, maintaining cellular homeostasis, and immune regulation [1,14,27,39,40]. The multiple roles of Hsp70s in various cellular events could account for their limited diversity. However, research on non-canonical Hsp70s remains scarce. From the limited studies available, it appears that non-canonical members are not constitutively expressed in animals, whereas constitutive members are shown to be more vital [27]. Given the diversity observed among the three non-canonical Hsp70 groups investigated in the current research, it is plausible that they are not constitutively expressed in cells either.

Up until now, only 41 species were confirmed to have all four types of full-length Hsp70s, and these species are dispersed throughout the animal kingdom (Table S2). This observation leads us to hypothesize that the majority of non-canonical Hsp70s have yet to be identified. Therefore, the current research serves as a systematic reference for future studies. Meanwhile, little research has explored the cellular and molecular functions of the three non-canonical Hsp70 groups that are the focus of this research. Although the roles of these non-canonical Hsp70s are probably not as crucial as those of cytosolic members, considering the conservativity and evolutionary relationships of the protein, further investigation into the functions of the non-canonical members could enhance our understanding of the relationships between Hsp70 structures, phylogenetic connections, and cellular roles.

## 4. Materials and Methods

### 4.1. Data Collection

Hsp70 family members were sourced from the databases of GenBank. To ensure a broad and comprehensive representation of phylogenetic diversity, members from each major group of Hsp70s were deliberately selected across various metazoan phyla (Table S1). Only full-length sequences were selected, while partial or putative sequences were not used. In cases where multiple nearly identical sequences in a single species were provided by different research groups, a representative sequence was chosen for inclusion in our analysis.

Through an extensive search of GenBank, 41 species that possessed all four types of Hsp70s were identified. Hsp70, Hsp70-12, Hsp70-13, and Hsp70-14 in each of these species were selected, and the corresponding open reading frames (ORFs) were collected (Supplementary Table S1).

### 4.2. Sequence Analysis

The molecular weights (Mws) and isoelectric point (pI) values of the collected ORFs were calculated by a Sequence Manipulation Suite Version 2 (SMS2) server (http://www.detaibio.com/sms2/, accessed on 28 March 2025). The corresponding values of different Hsp70 groups were presented with the Origin software package (version 9.8.0.200, Origin Lab, Northampton, MA, USA). The Turkey test was employed to examine the significance. *p* ≥ 5% was considered not significant. Shared letters denote groups not significantly different.

The 3D structure of the DmaHsp70s was predicted using the Alphafold3 online server (https://alphafoldserver.com/, accessed on 6 April 2025) with default settings. The simulation results were presented with PyMOL software (version 2.4, Schrödinger, New York, NY, USA). The PyMOL software was also applied to perform the structural alignments for each Hsp70 group using pairwise comparison. The results were presented using the default cartoon model, and different colors were used to mark individual amino acid sequences. The root-mean-square deviations (RMSDs) between 3D structures were calculated by VMD software (Version 2.0.0 Alpha).

### 4.3. Motif Identification and Presentation

To identify the locations of conserved motifs and amino acids in different Hsp70 groups, alignments were generated with Clustal Omega (http://www.clustal.org/, accessed on 30 March 2025) with default parameters. The alignments were presented by the DNAMAN9 software package (Lynnon Corporation, Pointe-Claire, QC, Canada). Prosite (https://prosite.expasy.org/ accessed on 30 March 2025) was employed to identify and present characterized motifs of acquired Hsp70s.

Full-length amino acid sequences of the canonical and non-canonical Hsp70s were used for identifying signature motifs. Five signature motif sequences of different Hsp70 groups were identified with the MEME program version 5.3.3 (https://meme-suite.org/meme/tools/meme, accessed on 31 March 2025), with default settings searching for ten motifs with a length of no more than twenty amino acids, and then further simplified. The corresponding motif logos were simulated in the MEME program and presented with Prosite as well.

The signature motif sequences identified were marked in the 3D structure of the four Hsp70s from the lamprey *Petromyzon marinus* because the species is a basal vertebrate representative used for motif mapping/comparative analyses.

### 4.4. Phylogenetic Analysis

A phylogenetic tree was constructed by MEGA 11.0 software (Mega Limited, Auckland, New Zealand,) using the neighbor-joining method with 1000 bootstrap replications, and a parallel analysis using the maximum likelihood method (1000 bootstrap replications) under the Jones–Taylor–Thornton (JTT) model with uniform rates among sites for cross-validation. A total of 172 full-length Hsp70 amino-acid sequences from different Hsp70 groups (Supplementary Table S2), including the Hsp70s marking the different lineages [13], were used to build the alignment. Only one canonical Hsp70 was used for building the tree (Supplementary Table S2). The phylogenetic tree was presented by FigTree v1.4 (tree.bio.ed.ac.uk).

### 4.5. Calculation of Synonymous and Nonsynonymous Substitution Rates

The representative ORFs of Hsp70, Hsp70-12, Hsp70-13, and Hsp70-14 from 41 species that possessed all four types of Hsp70s were selected. An alignment was performed to examine the orthology of the sequences before use. And all the sequences used were ORFs of the ones used for previous analysis and for building the phylogenetic tree. Codon-aligned nucleotide sequences of the ORFs were used for the analysis. The synonymous and nonsynonymous substitution rates were calculated using KaKs Calculator 2.0 [41] by performing both the Yang & Nielsen (YN) test and the maximum likelihood with weighted likelihood (MLWL) test (Supplementary Table S3). Both tests were used with an introduced gamma distribution and marked as gYN and gMLWL. A pairwise strategy was used for each sequence of the four Hsp70 groups. The inner- and inter-group Ka/Ks were calculated, respectively. The average Ka/Ks of different Hsp70 groups were presented by Origin, with an error bar represented by standard deviation. Brown–Forsythe test and Bartlett’s test were employed to examine the significance. *p* ≥ 5% was considered not significant. Shared letters denote groups not significantly different. Heat maps were created and presented by Origin software version 2021 (www.originlab.com/2021, accessed on 8 April 2025).

## Data Availability

Restrictions apply to the availability of these data. Data were obtained from NCBI and are available https://www.ncbi.nlm.nih.gov/#!/landingpage with the permission of [third party].

## Supplementary Materials

The following supporting information can be downloaded at https://www.mdpi.com/article/10.3390/ijms262311363/s1.

## Abbreviations

The following abbreviations are used in this manuscript:

Hsp70	Heat shock protein 70
ER	Endoplasmic reticulum
NBD	N-terminal nucleotide-binding domain
SBD	Substrate-binding domain
Mw	Molecular weights
pI	Isoelectric point
ORF	Open reading frames
RMSD	Root-mean-square deviations
